# Identification of a B-Cell Epitope in the VP3 Protein of Senecavirus A

**DOI:** 10.3390/v13112300

**Published:** 2021-11-18

**Authors:** Mi Chen, Lulu Chen, Jing Wang, Chunxiao Mou, Zhenhai Chen

**Affiliations:** 1 College of Veterinary Medicine, Yangzhou University, Yangzhou 225000, China; cm121321@163.com (M.C.); cll823207808@163.com (L.C.); wj229249720@163.com (J.W.); ytqxmcx@163.com (C.M.); 2 Joint International Research Laboratory of Agriculture and Agri-Product Safety, The Ministry of Education of China, Yangzhou University, Yangzhou 225000, China; 3 Jiangsu Co-Innovation Center for Prevention and Control of Important Animal Infectious Diseases and Zoonoses, Yangzhou University, Yangzhou 225000, China

**Keywords:** Senecavirus A, VP3 protein, B-cell epitope, monoclonal antibody

## Abstract

Senecavirus A (SVA) is a member of the genus *Senecavirus* of the family Picornaviridae. SVA-associated vesicular disease (SAVD) outbreaks have been extensively reported since 2014–2015. Characteristic symptoms include vesicular lesions on the snout and feet as well as lameness in adult *pigs* and even death in *piglets*. The capsid protein VP3, a structural protein of SVA, is involved in viral replication and genome packaging. Here, we developed and characterized a *mouse* monoclonal antibody (mAb) 3E9 against VP3. A motif ^192^GWFSLHKLTK^201^ was identified as the linear B-cell epitope recognized by mAb 3E9 by using a panel of GFP-tagged epitope polypeptides. Sequence alignments show that ^192^GWFSLHKLTK^201^ was highly conserved in all SVA strains. Subsequently, alanine (A)-scanning mutagenesis indicated that W193, F194, L196, and H197 were the critical residues recognized by mAb 3E9. Further investigation with indirect immunofluorescence assay indicated that the VP3 protein was present in the cytoplasm during SVA replication. In addition, the mAb 3E9 specifically immunoprecipitated the VP3 protein from SVA-infected cells. Taken together, our results indicate that mAb 3E9 could be a powerful tool to work on the function of the VP3 protein during virus infection.

## 1. Introduction

Senecavirus A (SVA) is a member of the genus *Senecavirus* of the family Picornaviridae, which is considered one of the etiological agents causing vesicular disease in *pigs*. The first isolated SVA strain, SVV-001, was discovered from a routine cell culture in 2002 [1]. Since the rapid emergence of SVA-associated vesicular disease (SAVD) in Brazil in 2014–2015 [2], SVA has become well known in the field of porcine diseases. Since then, SVA has been isolated from clinical samples of *pig* farms in the United States [3], Canada [4], Colombia [5], Vietnam [6], China [7], and Thailand [8]. As one of the etiological agents of swine vesicular disease, SVA can cause vesicular damage to the oral and nasal mucosa, lethargy, anorexia, lameness in *pigs*, and even acute death in *piglets* [9,10]. Because SVA induces similar vesicular disease as Foot and Mouth disease virus (FMDV), Swine vesicular disease virus (SVDV), and Vesicular stomatitis virus (VSV), it is difficult to distinguish them from each other in clinical practice.

SVA contains a single-stranded and positive-sense RNA genome, which encodes a polypeptide cleaved to a leader protein L and three precursor proteins P1, P2, and P3. Subsequently, P1 is cleaved into VP4, VP2, VP3, and VP1 structural proteins, while P2 and P3 are cleaved into 2A, 2B, 2C, 3A, 3B, 3C, and 3D nonstructural proteins [11]. Structural proteins are necessary to form a mature infectious viral particle. Nonstructural proteins are mainly involved in viral replication or gene expression regulation and do not bind to virions [12].

The picornavirus VP3 protein has been well investigated for its function. The VP3 protein of the Avian encephalomyelitis virus was reported to activate caspase-3-induced apoptosis [13]. The VP3 protein of the Duck hepatitis A virus plays an important role in the host’s cell adsorption and apoptosis [14]. FMDV VP3 evades the host innate immune system by inhibiting the IFN-β signaling pathway [15]. Maggioli et al. [16] found that SVA VP3 can elicit neutralizing antibodies at an early stage of virus infection. A recent study has shown that SVA VP3 protein is also involved in cell autophagy [17]. These findings indicate that picornavirus VP3 proteins play critical roles in the viral infection of the host.

Here, monoclonal antibodies (mAbs) of SVA VP3 were generated to investigate the distribution of VP3 in the SVA-infected cells, and a B cell epitope was finely mapped in this study. The detailed analysis of the epitope provides a deep understanding of the roles of amino acids in the epitope and viral replication. The results indicate that VP3-specific mAb will be useful in elucidating the function of VP3 during virus infection and developing new diagnostic reagents.

## 2. Materials and Methods

### 2.1. Virus, Cells and Animals

SVA GD05/2017 strain and its virus infectious clone plasmid (pC3-SVA-GD05) were kept at our laboratory. The ST-R, HEK293T, and BHK-21 cells were routinely cultured in Dulbecco’s modified Eagle’s medium (DMEM) containing 10% fetal bovine serum (FBS, Hyclone, Logan, UT, USA) at 37 °C in a 5% CO_2_ incubator. A SP2/0 myeloma cell line was passaged in RPMI-1640 supplemented with 20% heat-inactivated FBS. Female BALB/c *mice* were obtained from Yangzhou University Experimental Animal Center.

### 2.2. Expression, Purification and Identification of Recombinant VP3 Protein

A *VP3* gene fragment of the strain SVA GD05/2017 was amplified using primers VP3-F and VP3-R (Table S1), and the prokaryotic expression vector pCold II was used to express truncated SVA VP3 protein. The recombinant plasmid was transformed into *Escherichia coli* BL21 (DE3) cells and induced by 0.2 mM isopropyl β-D-thiogalactoside (IPTG) for 7–8 h at 16 °C. The bacterial cells were collected by centrifugation at 5000× *g* for 10 min. Then, the VP3 fusion proteins were identified by sodium dodecyl sulfate-polyacrylamide gel electrophoresis (SDS-PAGE). Recombinant VP3 protein (rVP3^pro^) was purified through Ni^2+^ column affinity chromatography and SDS-PAGE gel separation. The purified rVP3^pro^ was analyzed by SDS-PAGE and western blotting.

### 2.3. Preparation of Monoclonal Antibodies against VP3

Four female BALB/c *mice* (6-week old) were injected intraperitoneally with 10^8^ TCID50 SVA. The *mice* then received two more doses with 100 μg of purified rVP3^pro^ emulsified in Freund’s complete adjuvant and incomplete adjuvant at two weeks intervals, respectively. *Mice* with high antibody titers were lastly boosted by intraperitoneal injection with 50 μg rVP3^pro^ for hybridoma production. *Mice* were euthanized three days later, and spleen cells were obtained and then fused with SP2/0 cells by PEG 1500 (Solarbio, Beijing, China) as previously described [18]. The fused cells were plated into 96-well plates and grew in a hypoxanthine-aminopterin-thymidine (HAT) selection medium. Ten days later, the medium was changed to a hypoxanthine-thymidine (HT) medium. After HAT/HT medium selection, positive hybridoma cells were screened by indirect immunofluorescence assay (IFA) and subcloned 3–4 times by a limiting dilution method. Ascites fluid was obtained by intraperitoneal injection of positive hybridoma cells.

### 2.4. Characterization of the VP3 Monoclonal Antibody

The reactivity of mAb with rVP3^pro^ was analyzed by western blotting. Proteins were separated with 12% SDS-PAGE and blotted onto nitrocellulose membranes (Millipore Corp, Billerica, MA, USA). The membranes were blocked with 5% skim milk for 2 h. After washing three times with PBST, the membranes were then incubated with mAb of SVA-VP3 overnight at 4 °C. After washing, the membranes were further incubated with HRP-labeled goat anti-mouse IgG antibody (ABclonal Technology, Woburn, MA, USA) at 37 °C for 1 h. Following the washes, the membranes were treated with enhanced chemiluminescent (ECL) reagent (New Cell and Molecular Biotech, Suzhou, China) and visualized under the Tanon Chemiluminescent Imaging System (Biotanon, Shanghai, China).

Immunoprecipitation was performed as described below. Protein A/G immune precipitation beads (Bimake, Houston, TX, USA) were treated according to the manufacturer’s instructions and combined with mAb against SVA-VP3. SVA-infected ST-R cells (MOI = 0.01) were collected at 16–20 hpi, washed three times with PBS (pH 7.4), and suspended in immunoprecipitation (IP) lysis buffer containing 1 mM phenylmethylsulfonyl fluoride (PMSF) on ice for 10 min. The lysate supernatant was added to the beads-antibody mixture overnight at 4 °C. After washing the beads four times with lysis buffer, immunoprecipitated proteins were analyzed by western blotting using mAb 3E9, while the lysate from mock-infected ST-R cells was used as a control.

### 2.5. Identification of the Linear B-Cell Epitopes

To finely map the B-cell epitope of VP3-specific mAb, a panel of polypeptides was expressed according to previously described methods [19,20,21,22]. In the first round, five peptides spanning the SVA-VP3 protein (amino acids 123–157, 137–171, 161–195, 185–219, and 209–243) were expressed as GFP-fusion proteins. The gene fragments of desired peptide sequences were amplified and cloned into the pEGFP-C3 vector. The recombinant plasmids were transfected into HEK293T cells for protein expression using Lipofectamine 3000 Transfection reagent (Invitrogen, Waltham, MA, USA). Cells were collected at 24 h post transfection. Western blotting was used to confirm the expression of GFP-fused recombinant proteins with mAb 3E9. After that, a second-round panning was performed with five polypeptides spanning amino acids 161–219, which was expressed as GFP fusion proteins. The gene fragments of five polypeptides were cloned into pEGFP-C3, and the expression of recombinant proteins in HEK293T cells was identified by western blotting. In the last round, another panel of 11 peptides was produced by decreasing the number of amino acids one by one from the N-terminus and C-terminus of amino acids 186–201. Coding sequences were inserted into pEGFP-C3 and expressed as GFP fusion proteins. The primers are listed in Table S1.

### 2.6. Site-Directed Mutagenesis Assay

To identify amino acids that are critical for the epitope, each residue was sequentially substituted with alanine as described previously [23,24,25]. The primers used to introduce the specific mutations are shown in Table S2. Infectious clone pC3-SVA-GD05 was used as a template for site-directed mutagenesis, and ten mutants were constructed (G192A, W193A, F194A, S195A, L196A, H197A, K198A, L199A, T200A, and K201A). For PCR, the 50 μL reaction mixture was composed of 25 μL of PrimeSTAR Mix, 1 μL of forward primer, 1 μL of reverse primer, 21 μL of sterile dd H_2_O, and 2 μL of template. The PCR cycles were set as follows: predenaturation at 98 °C for 3 min, 98 °C for 10 s, 55 °C for 20 s, and 72 °C for 50 s, in 30–31 cycles., with the final extension at 72 °C for 8 min. The mutated fragments were digested with SacII and PpuMI and reintroduced into pC3-SVA-GD05. The mutant plasmids were transfected into BHK21 to rescue the viruses and were propagated blindly on ST-R for three generations. The rescued mutant viruses were subjected to IFA. For IFA, ST-R cells were infected with pC3-SVA-GD05 and the mutant viruses (multiplicity of infection [MOI] 0.01) and incubated for 16–24 h at 37 °C. After that, cells were washed twice with PBS, fixed with 4% formaldehyde for 10 min, and then permeabilized and blocked with 0.1% Triton-X-100 plus 2% BSA for 30 min. Cells were then rewashed and incubated with anti-VP3 or anti-3C mAbs (prepared in our lab) for 1 h in PBS. Cells were washed three times and incubated with Dylight 488 goat anti-mouse IgG antibody.

The *VP3* fragments were digested from the full-length clone pC3-SVA-GD05 and the mutants, by restriction enzymes KpnI and XhoI, and ligated into the plasmid pCAGGS-3xFLAG to generate pCAGGS-3FlLAG-VP3. ST-R cells were transfected with 2 μg of recombinant plasmids to express VP3 mutant proteins, which were analyzed using anti-FLAG or anti-VP3 as the primary antibody by Western blotting.

### 2.7. Bioinformatic Analysis

For sequence alignment, all SVA strains available from GenBank were analyzed using the MAFFT software to investigate the conservation of the VP3 epitope. The SVA-VP3 amino acid sequence was imported into the SWISS online system to predict the structure of VP3. Based on the results obtained from the SWISS-model online server, the epitope position was mapped to the three-dimensional (3D) structural model of SVA-VP3 using PyMOL software, and the position was indicated by colors to distinguish the location.

## 3. Results

### 3.1. Expression, Purification, and Identification of Recombinant VP3 Protein

**Figure 1:**
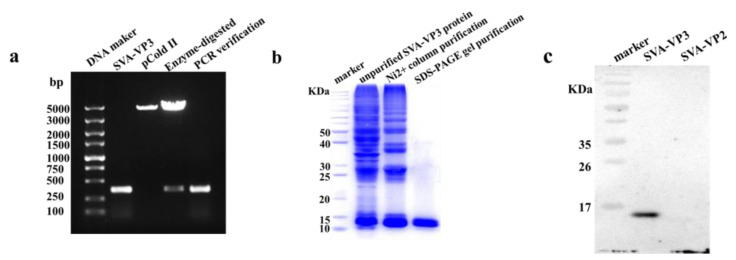
Expression and purification of recombinant SVA-VP3 protein. (**a**) The *SVA-VP3* gene was amplified by RT-PCR and inserted into a pColdII vector. The pColdII-SVA-VP3 was identified by restriction enzyme digestion and PCR. (**b**) Recombinant SVA-VP3 protein was purified through Ni^2+^ column affinity chromatography and SDS-PAGE gel purification. All protein samples were analyzed by SDS-PAGE. (**c**) The recombinant SVA-VP3 protein was identified by Western blotting using an anti-His-tag antibody. Recombinant SVA-VP2 protein was used as a negative control.

To express the SVA-VP3 protein, the *VP3* gene was amplified and cloned into a pCold II vector. The recombinant plasmid pCold II-VP3 was verified by restriction enzyme digestion with KpnI and EcoRI and PCR amplification. The results showed that the correct DNA bands were obtained from the recombinant plasmid (Figure 1a).

The plasmid pCold II-VP3 was then transformed into BL21(DE3) cells, and recombinant VP3 protein with a His-Tag was successfully expressed (Figure 1b). The molecular weight of VP3 protein was approximately 13.5 kDa. The target protein expressed in the form of inclusion body was purified through Ni^2+^ column affinity chromatography and SDS-PAGE gel purification. The purified recombinant VP3 protein was identified by Western blotting with an anti-His tag antibody (Figure 1c).

### 3.2. Generation and Characterization of mAbs against VP3

**Figure 2:**
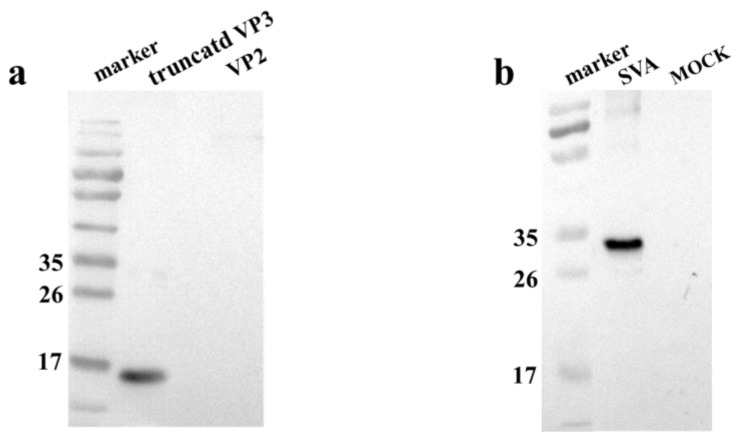
Identification of VP3 monoclonal antibody; (**a**) Characterization of mAb by Western blotting. The VP3 proteins expressed in *E. coli* BL21(DE3) were collected for SDS-PAGE, and the VP2 protein expressed in *E. coli* BL21(DE3) was used as a control group. (**b**) Immunoprecipitation of VP3 protein in the SVA-infected ST-R cells. The mAb 3E9 was used as the primary antibody, and the horseradish-peroxidase-labeled goat anti-mouse IgG antibody was used as a secondary antibody.

The purified rVP3^pro^ was used to immunize *mice* for the preparation of mAbs against VP3. One hybridoma cell line secreting antibody specific to SVA VP3 protein was selected and subcloned four times by limiting dilution. It was named mAb 3E9 and reacted specifically with the rVP3^pro^ expressed in bacterial cells (Figure 2a) detected by Western blotting, with the molecular weight of rVP3^pro^ of about 13.5 kDa. The mAb 3E9 can also immunoprecipitate native SVA VP3 protein (26.8 kDa) expressed in the SVA-infected ST-R cells (Figure 2b). These results demonstrated that mAb specifically recognized the SVA VP3 protein.

### 3.3. Identification of the Minimal Epitope

**Figure 3:**
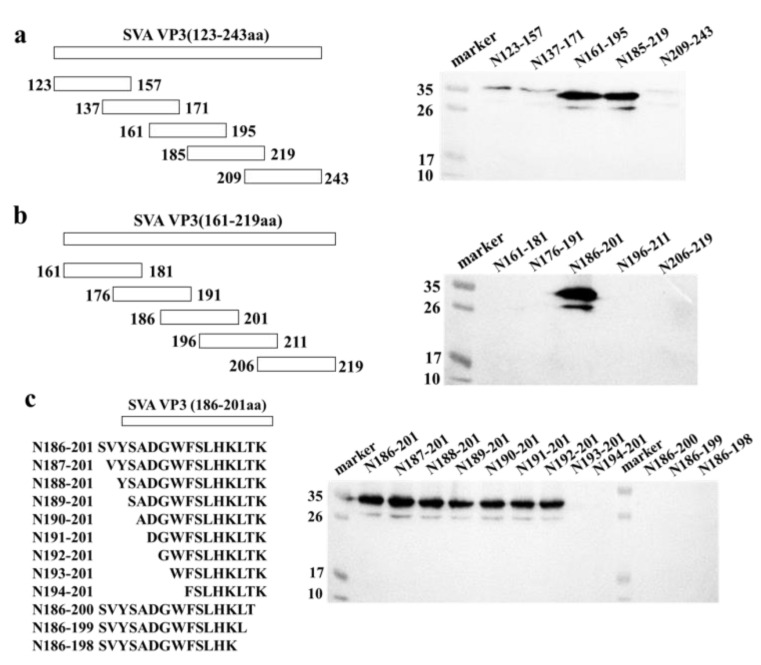
Epitopes mapping by Western blotting; (**a**) The SVA-VP3 protein (123–243aa) was truncated into five segments with 10-amino acid overlapping between neighboring parts and subjected to Western blotting with mAb 3E9. (**b**) The fragment of SVA-VP3 N161-219 was divided into five peptides with 5-amino acid overlapping and subjected to Western blotting with mAb 3E9. (**c**) A panel of truncated peptides covering the whole SVA-VP3 N186-201 fragment were expressed and subjected to Western blotting to determine the minimal epitope of mAb 3E9.

To roughly identify the epitopes, five overlapping fragments of *VP3* were expressed in eukaryotic cells. Western blotting results show that the mAb 3E9 only reacted with N161-195 and N185-219 rather than N123-157, N137-171, or N209-243, indicating that the epitopes may be located in the N161-219 fragment (Figure 3a). To further determine the epitope, another five fragments of VP3 were expressed. The results showed that mAb 3E9 only reacted with N186-201, indicating a smaller epitope located in the N186-201 fragment (Figure 3b). In the last round, another panel of 11 peptides with distinct numbers of amino acids was expressed and subjected to Western blotting. As shown in Figure 3c, the common part ^192^GWFSLHKLTK^201^ was the minimal epitope.

### 3.4. Reactivity of the mAb with Different SVA Strains

**Figure 4:**
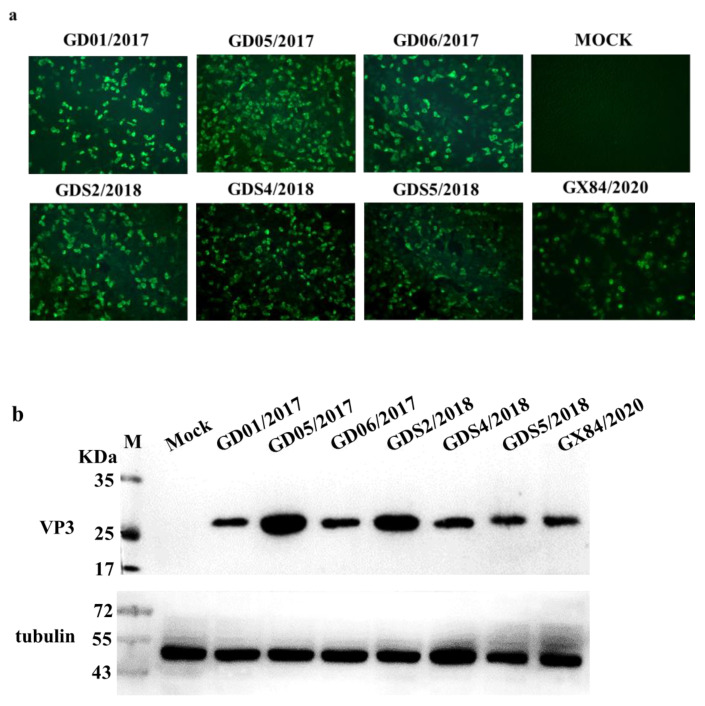
Identification of Reactivity of mAb 3E9 to different SVA strains by IFA (**a**) and Western blot (**b**). The reactivity of mAb 3E9 to ST-R cells infected by different SVA strains was analyzed. Normal ST-R cells were used as a negative control.

IFA was carried out to determine whether mAb 3E9 could react with different SVA strains. ST-R cells were infected with ten SVA strains that were stored in our laboratory. Using mAb 3E9 as a primary antibody, IFA showed that mAb 3E9 had good reactivity with all seven SVA strains (Figure 4).

### 3.5. Amino Acid Alignment of Epitopes

**Figure 5:**
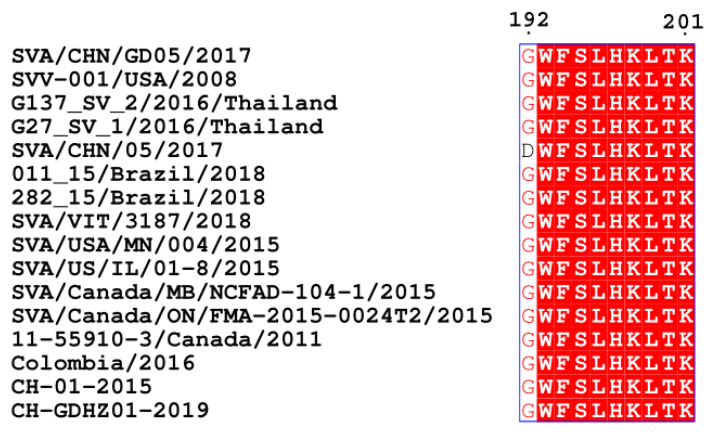
Comparison of the ^192^GWFSLHKLTK^201^ epitope amino acid sequence among different SVA strains. All SVA strains from GenBank were selected for sequence alignment using MAFFT. Sequences of representative strains are shown.

To explore the level of conservation of the VP3 epitope, all SVA strains from GenBank were selected for sequence alignment using MAFFT. The results indicated that the epitope ^192^GWFSLHKLTK^201^ was highly conserved in the VP3 protein among SVA strains, except for an Asp to Gly substitution at position 192 in the strain SVA/CHN/05/2017 (Figure 5).

**Figure 6:**
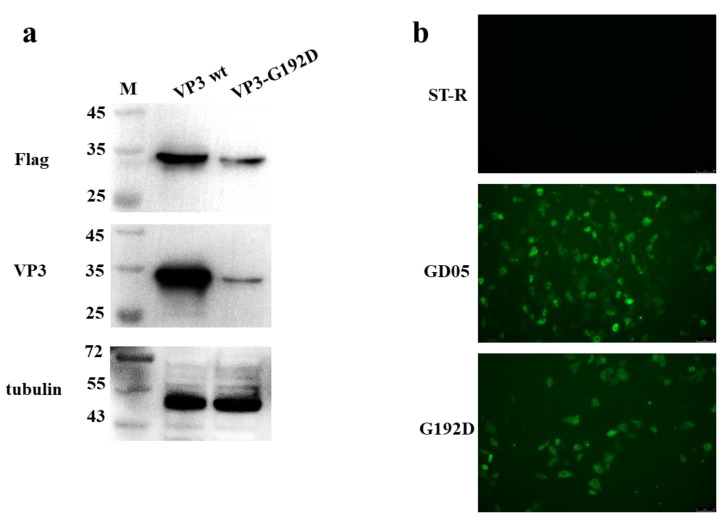
Identification of the reactivity between mAb 3E9 and the mutant epitope of SVA/CHN/05/2017 strain. (**a**) ST-R cells were transfected with pCAGGS-3FlLAG-VP3 and pCAGGS-3FLAG-VP3-G192D for 48 h and then immunoblotted with anti-FLAG and anti-VP3 mAbs. (**b**) ST-R cells were infected with SVA/GD05/2017 and SVA/CHN/05/2017(G192D), respectively. IFA was performed using anti-VP3 mAb.

To determine if mAb 3E9 could react with the mutant epitope of SVA/CHN/05/2017 strain, infectious clone pC3-SVA-GD05 was used as a template for site-directed mutagenesis, and the position Gly^192^ was substituted with an Asp. The rescued virus G192D was subjected to IFA, and its VP3 protein was subjected to WB using mAb 3E9. As shown in Figure 6a,b, mAb 3E9 recognized the mutant epitope of the SVA/CHN/05/2017 strain.

### 3.6. Spatial Distribution of the Novel Epitope

**Figure 7:**
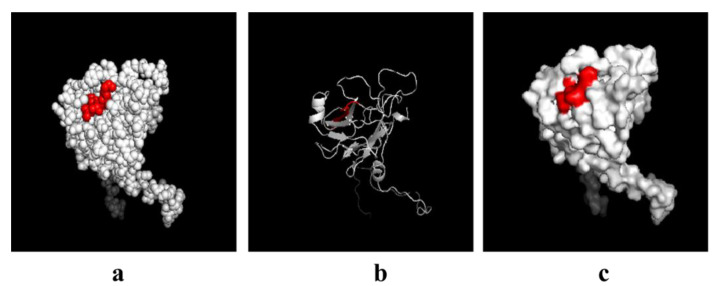
Spatial distribution of the identified epitope on the VP3. The relative spatial position of the identified epitopes is presented in spheres (**a**), and cartoon (**b**) from the predicted three-dimensional structure of SVA VP3, and the epitope recognized by VP3 is shown in red. The epitope is located on the surface of the protein (**c**).

The three-dimensional structure of SVA VP3 protein was constructed by the SWISS online system. Furthermore, the location of the VP3 epitope was analyzed by PyMOL software. As shown in Figure 7, the epitope was located on the surface of the VP3 protein.

### 3.7. Identification of the Critical Amino Acid Residues of the Epitope

**Figure 8:**
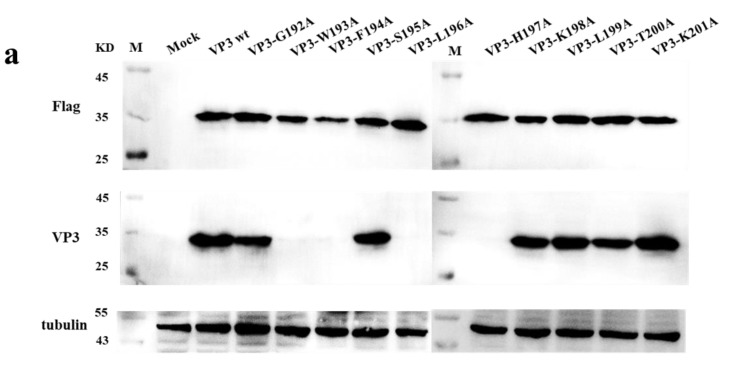

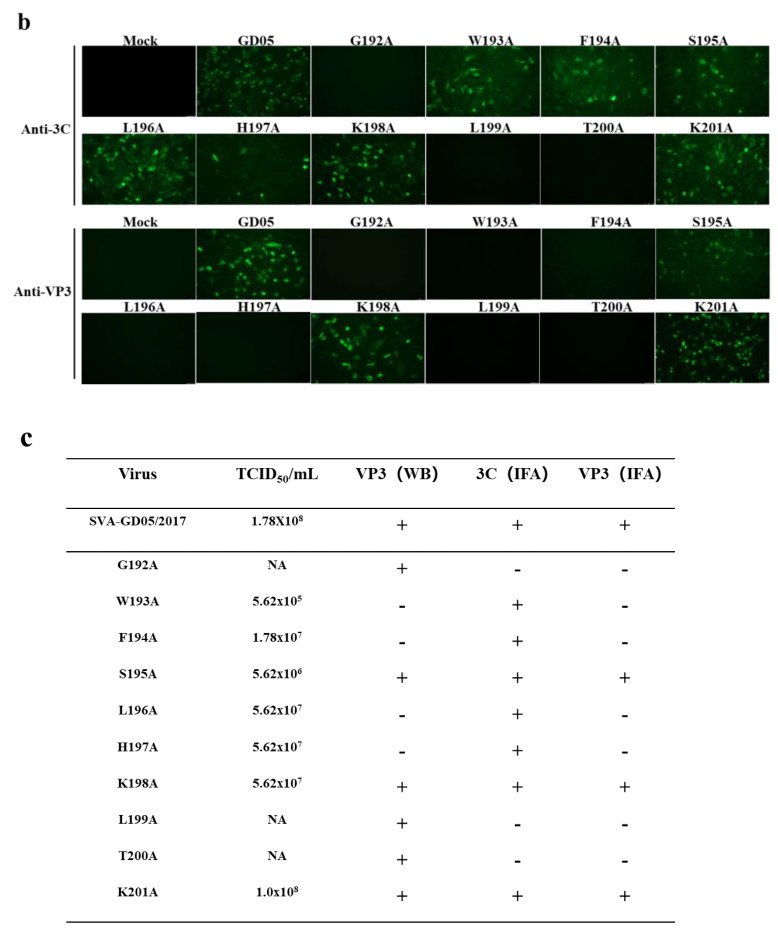
Alanine-scanning mutagenesis. (**a**) Alanine-scanning mutagenesis to determine the critical amino acids of the VP3 epitope ^192^GWFSLHKLTK^201^. ST-R cells were transfected with pCAGGS-3FLAG-VP3 mutants for 48 h and then immunoblotted with anti-FLAG and anti-VP3 mAbs M: marker. (**b**) A group of pC3-SVA-GD05 plasmids was used to rescue the recombinant viruses, which were then propagated in the ST-R cells for three generations and analyzed by IFA using anti-VP3 mAbs. (**c**) Identification of SVA mutant viruses by virus rescue and immunological investigation with mAb 3E9. ‘NA’ means not available, ‘+’ means positive, ‘-’ means negative.

To further delineate the residues contributing to the activity of the ^192^GWFSLHKLTK^201^ epitope, a group of mutant viruses were modified, in which each residue of the 10-aa peptide was substituted in turn by alanine. All mutant viruses were rescued, except G192, L199, and T200, suggesting that sites 192, 199, and 200 of VP3^pro^ were essential for SVA replication (Figure 8b,c). The reactivity of the mutant viruses to mAb 3E9 was detected by IFA (Figure 8b,c). IFA results showed that the W193A, F194A, L196A, and H197A mutations completely abrogated the reactivity of the epitope with mAb 3E9. Similarly, the reactivity of the mutant VP3 proteins to mAb 3E9 was detected by Western blotting (Figure 8a,c), which indicated that the residues Trp^193^, Phe^194^, Leu^196^, and His^197^ were critical binding sites for mAb 3E9. However, the substitution of the Gly^192^, Ser^195^, Lys^198^, Leu^199^, Thr^200^, and Lys^201^ with alanine had no effect on the reactivity between the epitope and mAb 3E9.

## 4. Discussion

SVA-associated vesicular disease (SVAD) has been reported in many countries since 2014–2015 [2,3,4,5,6,7,8]. The outbreak of the emerging infectious disease has resulted in economic losses to the *swine* industry. Unfortunately, there is no commercial SVA vaccine for *pigs*, though recombinant live vaccine candidate strains [26] and an oil adjuvant inactivated vaccine candidate [27] have been reported. Therefore, it remains essential to monitor the virus epidemic and investigate the antigenicity of virus field strains. Preparation of mAbs against SVA contributes to identifying viral antigen epitopes, which will provide a powerful tool for structure and function research on viral proteins and develop new diagnostic reagents.

So far, the reported mAbs against SVA were designed against viral particles and the VP1 and VP2 proteins. Yang et al. immunized *mice* with binary ethylenimine (BEI)-inactivated SVA to produce five mAbs and developed an SVA-specific competitive enzyme-linked immunosorbent assay (cELISA) for serodiagnosis. Fan et al. prepared eight monoclonal antibodies against VP1 and VP2 by using recombinant VP1 and VP2 proteins. Six epitopes against these mAbs were first revealed, including ^21^GELAAP^26^ on VP1 and ^12^DRVITQT^18^, ^71^WTKAVK^76^, ^98^GGAFTA^103^, ^150^KSLQELN^156^, and ^248^YKEGAT^253^ on VP2 [28]. SVA VP3 protein is located on the surface of the virus and forms the viral capsid, which is an important structural protein involved in viral replication. Here, we expressed truncated SVA VP3 via a prokaryotic expression system and prepared a specific monoclonal antibody against VP3. *Mice* were immunized with prokaryotic-expressed VP3 every two weeks. Finally, one hybridoma cell line that secreted SVA VP3 monoclonal antibody was obtained after cell fusion, positive screening, and subcloning. We used the IFA method to detect positive hybridomas with SVA-infected ST-R cells, which can effectively eliminate the problem of false positive antibodies.

Epitopes are also known as antigenic determinants, which are composed of specific amino acid or chemical groups on the antigen molecule that determine the specificity of the antigen. Epitope identification includes epitope peptide scanning, protein cleavage, and phage display technology [29,30]. In this study, the eukaryotic expression vector pEGFP-C3 was used to express truncated VP3 protein to identify epitopes. This system is not only simple to operate but can also accurately identify the epitopes. The pEGFP-C3 carries a green, fluorescent label, and the expression of the fusion protein can be directly observed under an inverted fluorescence microscope. The epitope ^192^GWFSLHKLTK^201^ was identified by Western blotting.

To investigate the applicability of the mAb 3E9, ST-R cells were infected with seven SVA strains kept in our laboratory. IFA showed that the mAb 3E9 could specifically react with all of them. Comparative analysis of SVA VP3 sequences available from GenBank indicated that the epitope sequence of ^192^GWFSLHKLTK^201^ was highly conserved among almost all of the SVA strains, except SVA/CHN/05/2017 with one amino acid mutation. Subsequent analysis showed that the mAb 3E9 could react with the mutant epitope of strain SVA/CHN/05/2017. Furthermore, other SVA strains that contain the ^192^GWFSLHKLTK^201^ epitope can be recognized by mAb 3E9, indicating that the monoclonal antibody has the potential to react with all known SVA strains.

These findings provided useful information for understanding the antigenicity of VP3 and may be valuable in developing epitope-based vaccines or diagnostic kits for SVA infection. In addition, to identify the critical amino acid residues of the epitope, a series of mutant viruses were constructed by reverse genetics. As shown in Figure 8, three mutant viruses (G192A, L199A, and T200A) could not be rescued, but mutations at these three sites did not affect the reaction between VP3 protein and mAb 3E9, suggesting that amino acid residues at the sites 192,199, and 200 of VP3 were necessary for SVA replication but did not affect the antigenicity of the VP3 protein. The W193A, F194A, L196A, and H197A mutations were successfully rescued, and the results from Western blots and IFA showed that they completely abrogated the reactivity of VP3 protein with mAb 3E9 (Figure 8b,c), suggesting that the four amino acids were critical residues on this linear epitope. These results may provide new insights into the antigenicity of SVA. Furthermore, the results showed that the S195A, K198A, and K201A mutations did not affect either viral replication or the antigenicity of the VP3 protein.

## 5. Conclusions

In summary, a novel monoclonal antibody of SVA was obtained in this study, and a linear B cell epitope against SVA VP3 was identified for the first time, which provides a powerful immunological tool to elucidate the function of the VP3 protein during virus infection as well as develop new diagnostic reagents.

## Data Availability

The data that supports the findings of this study are available from the corresponding author, Z.C., upon reasonable request.

## Supplementary Materials

The following are available online at https://www.mdpi.com/article/10.3390/v13112300/s1, Table S1: Primers used for construction of VP3 truncations, Table S2: Primers used to generate specific mutations in the VP3 gene of the infectious clone pC3-SVA-GD05.
