# Supplementary material for: Identification of a B-Cell Epitope in the VP3 Protein of Senecavirus A
Source: Viruses. 2021 Nov 18;13(11):2300. doi: 10.3390/v13112300 (PMC8621820; doi:10.3390/v13112300)
Supplement: Supplementary file 1 [file viruses-13-02300-s001.zip › viruses-1441625-supplementary.pdf]

APPLIED MICROBIOLOGY AND BIOTECHNOLOGY

Identification of a B-cell epitope in the VP3 protein of Senecavirus A

Mi Chen[a], Lulu Chen[a], Jing Wang[a], Chunxiao Mou[a], Zhenhai Chen[a,b,c #]

[a]College of Veterinary Medicine, Yangzhou University, Yangzhou, JS, China;

[b]Joint International Research Laboratory of Agriculture and Agri-Product Safety, the Ministry of Education of China, Yangzhou University, China;

[c]Jiangsu Co-Innovation Center for Prevention and Control of Important Animal Infectious Diseases and Zoonoses, Yangzhou University, China.

[#]corresponding author

Corresponding author:

Zhenhai Chen, Ph.D. | zhenhai@yzu.edu.cn

Professor, College of Veterinary Medicine

Yangzhou University

12 Wen-hui East Road

Yangzhou, JS225009, China

Cell:18252747459

Tel: +86-514-89798271

Fax: 0514-87972218

Table S1

Primers used for construction of VP3 truncations.

Name		Sequence(5′-3′)	Residues sites
VP3	F	TGCCGGGGTACCGGACCTATGATGGCAAGA	123-243aa
	R	GCACCGGAATTCTTAGTTGTCGGTGGAGTGGAA	
N123-157	F	CAGAACGAGCTCGGACCTATGATGGC	123-157aa
	R	CAGCACCCGCGGGTTTAAGCCTATATCC	
N137-171	F	CATGACGAGCTCCAACCACAGACCCTTTC	137-171aa
	R	CAATACCCGCGGCCGAGTTTCAC	
N161-195	F	CAGCACGAGCTCTCTAGTTGGACCTTTG	161-195aa
	R	CAGCTCCCGCGGAATTTTGGTCAGC	
N185-219	F	CATCAGAGCTCATTACCAATTCGG	185-219aa
	R	CAATACCCGCGGGGTGTAATCC	
N209-243	F	CAGCAGAGCTCCTACCACCTGATTGC	209-243aa
	R	CAATACCCGCGGGTTGTCGGTG	
N161-181	F	CAGCAGAGCTCAGTTGGACCTTTGTCATC	161-181aa
	R	CAATACCCGCGGCCGAGTTTCACGGTAATC	
N176-191	F	CAGCAGAGCTCTACCGTGAAACTCGGG	176-191aa
	R	CAATACCCGCGGATCAGCAGAATAAACTGAG	
N186-201	F	CAGCAGAGCTCGTTTATTCTGCTGATGG	186-201aa
	R	CAATACCCGCGGTTTGGTCAGCTTGTG	
N196-211	F	CAGCAGAGCTCCACAAGCTGACCAAAATTC	196-211aa
	R	CAATACCCGCGGACTCTGTGGGCAG	
N1206-219	F	CAGCAGAGCTCCCTGACTGCCCACAG	206-219aa
	R	CAATACCCGCGGAGAGGCAAAAAAGAG	

Table S2

Primers used to generate specific mutations in the VP3 gene of the infectious clone pC3-SVA-GD05.

Mutant		Sequence(5′-3′)
G192A	F	CCAACTCAGTTTATTCTGCTGATgctTGGTTTAGCCTGCACAAGCTG
	R	CAGCTTGTGCAGGCTAAACCAagcATCAGCAGAATAAACTGAGTTGG
W193A	F	AACTCAGTTTATTCTGCTGATGGTgctTTTAGCCTGCACAAGCTGAC
	R	TAATTTTGGTCAGCTTGTGCAGGCTAAAagcACCATCAGCAGAATA
F194A	F	TTATTCTGCTGATGGTTGGgctAGCCTGCACAAGCTGACCAAAATT
	R	TTTGGTCAGCTTGTGCAGGCTagcCCAACCATCAGCAGAATAAAC
S195A	F	ATTCTGCTGATGGTTGGTTTgctCTGCACAAGCTGACCAAAATTACT
	R	TTGGTCAGCTTGTGCAGagcAAACCAACCATCAGCAGAATAAACT
L196A	F	GCTGATGGTTGGTTTAGCgctCACAAGCTGACCAAAATTACTCTAC
	R	TAATTTTGGTCAGCTTGTGagcGCTAAACCAACCATCAGCAGAATAAA
H197A	F	CTGCTGATGGTTGGTTTAGCCTGgctAAGCTGACCAAAATTACTC
	R	GAGTAATTTTGGTCAGCTTagcCAGGCTAAACCAACCATCAGCAG
K198A	F	TGATGGTTGGTTTAGCCTGCACgctCTGACCAAAATTACTCTACCAC
	R	TGGTAGAGTAATTTTGGTCAGagcGTGCAGGCTAAACCAACCATCAGC
L199A	F	TTGGTTTAGCCTGCACAAGgctACCAAAATTACTCTACCACCTGAC
	R	TGGTAGAGTAATTTTGGTagcCTTGTGCAGGCTAAACCAACCATC
T200A	F	GGTTGGTTTAGCCTGCACAAGCTGgctAAAATTACTCTACCACC
	R	GGTGGTAGAGTAATTTTagcCAGCTTGTGCAGGCTAAACCAACC
K201A	F	TTTAGCCTGCACAAGCTGACCgctATTACTCTACCACCTGACTGC
	R	GTCAGGTGGTAGAGTAATagcGGTCAGCTTGTGCAGGCTAAACCAAC
